# Mucosal administration of lipid nanoparticles containing self-amplifying mRNA induces local uptake and expression in a pig model as a potential vaccination platform against STIs

**DOI:** 10.1007/s13346-025-01877-x

**Published:** 2025-06-11

**Authors:** Ibe Van de Casteele, Magalie Plovyt, Magdalena Stuchlíková, Michiel Lanssens, Ben Verschueren, Quenten Denon, Paul Van der Meeren, Sean McCafferty, Arlieke Gitsels, Pieter Cornillie, Niek N. Sanders, Aster Vandierendonck, Katrien C. K. Poelaert, Daisy Vanrompay

**Affiliations:** 1 Ziphius NV, B-9052 Zwijnaarde, Belgium; 2 Laboratory for Immunology and Animal Biotechnology, Department of Animal Sciences and Aquatic Ecology, Faculty of Bioscience Engineering, Ghent University, B-9000 Ghent, Belgium; 3 Particle and Interfacial Technology Group (PainT), Department of Green Chemistry and Technology, Faculty of Bioscience Engineering, Ghent University, B-9000 Ghent, Belgium; 4 Laboratory of Gene Therapy, Department of Veterinary and Biosciences, Faculty of Veterinary Medicine, Ghent University, B-9820 Merelbeke, Belgium; 5 Laboratory of Veterinary Morphology, Department of Morphology, Imaging, Orthopedics, Rehabilitation and Nutrition, Faculty of Veterinary Medicine, Ghent University, B-9820 Merelbeke, Belgium

**Keywords:** Self-amplifying mRNA—Lipid nanoparticles, Mucosal administration, Mucosal spray, Pig model, Sexually transmitted infections

## Abstract

Mucosal vaccination generates protective immune responses directly at the primary site of STI infection. However, the delivery of nanoparticles is hindered by the mucus barrier at these mucosal surfaces. Due to this interference, research on mucosal administration of self-amplifying (sa)-mRNA encapsulated in lipid nanoparticles (LNP) is currently limited and inconsistent. Some progress has been reported for nasal mRNA vaccination. However, for STIs, protective immune responses are required at the urogenital tract, which is achieved through intravaginal or intranasal administration. Therefore, in this research, we aimed to determine whether an sa-mRNA-LNP reporter vaccine could be effectively administered mucosally, evaluating its potential as a novel platform for STI vaccination. The sa-mRNA luciferase construct was encapsulated in two LNP formulations. In vitro studies demonstrated that these formulations maintained their potency after being sprayed with different sprayers and exposed to different mucus solutions, except for a human cervicovaginal simulant. Next, pigs received 15 µg of the sa-mRNA intravaginally and intranasally through a mucosal spray or injection. The mucosal spray resulted in expression and uptake only at the vaginal mucosa, whereas injection of the formulations resulted in expression at both mucosal sites. However, expression after spraying in the vaginal mucosa disappeared by day 4 post-administration. No differences were observed between both LNP formulations. These findings demonstrate that sa-mRNA can be used for mucosal administration, and expression can be achieved in a more relevant animal model. However, additional research is needed to develop more suitable particles for these complex environments.

**Supplementary Information:**

The online version contains supplementary material available at 10.1007/s13346-025-01877-x.

## Introduction

Sexually transmitted infections or “STIs” can have a severe impact on sexual and reproductive health in humans. Some of the most common STIs include *Chlamydia, Syphilis, Gonorrhea,* and *Trichomoniasis*. In 2020, the World Health Organization (WHO) estimated 374 million new infections with one of these four pathogens [1]. Despite public awareness and the availability of preventatives and cures, the incidence of STIs continues to rise. In Europe, an increase of 48% for *Gonorrhea*, 34% for *Syphilis* and 16% for *Chlamydia* has been reported in 2022 [2].

As many of these infections start asymptomatically, a preventive vaccine would be the best solution to combat the incidence of STIs and their adverse outcomes. Although considerable effort has already been invested in STI vaccine development, there are still significant challenges such as difficulties in eliciting the necessary immune responses. Lessons learned from past vaccination trials suggest that mucosal vaccination could be the key to developing effective STI vaccines [3–5]. The added benefit of mucosal vaccination lies in its unique capability to generate protective mucosal IgA responses and tissue-resident memory cells at the site of infection [6]. These local immune responses can be generated not only by reproductive tract vaccination but also by vaccinating at distal mucosal sites, such as the nasal cavity. This concept is known as the common mucosal immune system [7], which enables human vaccination at a more widely accepted site than the reproductive tract.

Since mucosal sites are at the critical interface between the external environment and the underlying tissues of the host, they contain several protective barriers [8]. Inconveniently, these barriers also interfere with the development of mucosal vaccines. One such barrier is the tolerogenic barrier. Due to constant exposure to foreign material, mucosal surfaces contain many regulatory T-cells to prevent excessive inflammation and overreaction of the immune system to these foreign and often harmless particles [9, 10]. To overcome this barrier, a strong mucosal adjuvant is required to enable the induction of mucosal immune responses. However, the options for safe and effective mucosal adjuvants are currently limited, complicating the development of mucosal subunit vaccines [11].

One potential solution to overcome the tolerogenic mucosal barrier is the use of self-amplifying (sa)-mRNA vaccines, as they are reported to possess an inherent self-adjuvating effect through the amplification step [12, 13]. As such, sa-mRNA vaccines have been described to offer increased immunogenicity at lower doses compared to non-replicating mRNA [14]. Despite these potential advantages, research involving mucosal sa-mRNA administration is limited. To our knowledge, intravaginal sa-mRNA administration has never been reported, while intranasal sa-mRNA vaccination has only been described in two studies. These studies report contradictory results, with Jennewein et al. [15] indicating strong systemic and local mucosal immune responses in the lungs of mice, while Anderluzzi et al*.* [16] demonstrated weak responses in a similar setting using four different nanoparticles.

One possible explanation for these discrepant results is the other important barrier at the mucosal surfaces, the mucus barrier. Indeed, mucosal surfaces are covered by a mucus layer, which consists of water, proteins, lipids, salts, and cellular debris. The main component of mucus is the negatively charged, highly glycosylated mucin polymers, which form a mesh structure. These polymers can interact with nanoparticle formulations, facilitating their removal through mucociliary clearance. To avoid interactions between nanoparticles and mucus and improve delivery, nanoparticles can be adjusted to increase their mucopenetrative or mucoadhesive properties [17, 18]. However, at the moment, it is not clear whether LNPs are suited for mucosal delivery, and which LNP properties can improve mucosal delivery.

Another significant limitation in the field of mucosal delivery is the reliance on animal models. The translatability of the mouse model for human intranasal application is limited due to anatomical and immunological differences. On the contrary, the larger pig model would be more suited as they have a Waldeyer’s ring, an anatomically comparable nasal cavity, and a similar microbiome to humans. Additionally, pigs consist of an outbred population [19]. Due to its comparable size and anatomy, the pig model can be used to test needle-free administration methods such as a mucosal spray. Since mucosal tissues are highly sensitive, needle-free administration methods are needed. Mucosal spraying of therapeutics is also more widely accepted in society compared to the nasal drops used in mouse models.

Therefore, we investigated whether nasal and vaginal mucosal administration of an sa-mRNA-LNP reporter construct using mucosal spraying is feasible in a pig model. We aimed to demonstrate the local uptake and expression in the treated tissues and at the draining lymph nodes. Therefore, an sa-mRNA reporter construct encoding firefly luciferase was encapsulated in two different fluorescently labeled lipid nanoparticles (LNPs). LNP 1 was a standard C12-200-based LNP formulation optimized for mRNA delivery by Kauffman et al. [20]. To enhance sa-mRNA delivery, this formulation was further optimized in-house, resulting in LNP 2. Both LNP formulations were analyzed to increase the likelihood of success.

## Material and methods

### Sprayer droplet size measurements

Droplet size analysis of three different sprayers was conducted to select the most optimal sprayer for the in vivo trial. The assessed sprayers included the MADgic Laryngotracheal Mucosal Atomization Device (Teleflex, Athlone, Ireland), a PennCentury model IA-1 C (PennCentury, Philadelphia, PA, USA), and a sprayer (model A1) developed by a local precision mechanics company (Verbeeken Precisiemechaniek, Oostkamp, Belgium). The latter was based on the design of the PennCentury model IA-1 C.

Droplet size measurements were performed using a Mastersizer S (Malvern Panalytical, Malvern, UK). A lab Alliance series ii HPLC pump (Waters, Milford, MA, USA) facilitated the delivery of a sufficient volume of water at a constant flow rate for efficient droplet size measurement. The pump was tested at a flow rate of 8 mL/min and 10 mL/min, directing the sprayed particle cloud towards the center of the laser beam of the Mastersizer S. Each measurement comprised three repetitions. The refractive index of the medium was set to 1.00 (air), while that of the droplets was set to 1.33 (water). An absorption factor of 0 was used and the obscuration level varied from 0.2 to 27.0%, depending on the droplet size. Subsequently, the data were processed using a polydisperse model in the Mastersizer software v2.15 (Malvern Panalytical).

### In vitro screening of lipid nanoparticles (LNPs)

#### Self-amplifying (sa-)mRNA production, purification, and quality control

The plasmid used for sa-mRNA production contains a T7 RNA polymerase promotor sequence followed by the sa-mRNA sequence based on the TC-83 vaccine strain of the Venezuelan Equine Encephalitis Virus (VEEV). In the Non-Structural Protein (NSP) sequence, the following mutations were implemented: A3 > G substitution to increase resistance against type I IFNs and the non-cytopathic NSP2.Q739L mutation to enhance replication and reduce cytotoxicity [21, 22]. As the gene of interest (GOI), a codon optimized firefly luciferase sequence (GenBank: AY738222) was inserted as a reporter protein in the sa-mRNA sequence. I-Sce-I restriction sites flanked the sa-mRNA sequence for linearization.

The plasmid was inserted inside DH5α competent *Escherichia coli* (*E. coli*, New England Biolabs (NEB), Ipswich, MA, USA) through heat shock transformation. Next, the plasmid DNA was purified from *E. coli* using a plasmid plus midi kit (Qiagen, Hilden, Germany) according to the manufacturer's guidelines. The plasmid was linearized using the I-Sce-I restriction enzyme (NEB) and the linearized plasmid was purified using the Wizard SV gel and PCR clean-up system (Promega, Madison, WI, USA). Next, the sa-mRNA was produced using the HiScribe T7 high-yield RNA synthesis kit (NEB) and CleanCap AU (TriLink BioTechnology, San Diego, CA, USA) for co-transcriptional capping. Following the in vitro transcription reaction, a silica purification was performed using the Monarch RNA cleanup kit (NEB) and a final cellulose purification was performed to remove double-stranded RNA by-products as described in the publication of Baiersdörfer et al*.* [23]. Briefly, a chromatography buffer was used to suspend cellulose (Avicel PH-101 microcrystalline cellulose, Sigma-Aldrich, St. Louis, MO, USA) at a concentration of 0.2 g cellulose/mL. Afterwards, 700 µL of the cellulose slurry was placed in a spin column (0.45 µm cut-off, Millipore, Burlington, MA, USA) and centrifuged at 14,000 RCF for 1 min. The cellulose was then washed with chromatography buffer before adding the sa-mRNA. After centrifugation, 500 µg of sa-mRNA in chromatography buffer was added, which was shaken at maximum speed with the cellulose for 30 min. After centrifugation at 14000 RCF, the flow-through contained the single-stranded sa-mRNA. To ensure excellent purity, the sa-mRNA was purified once more with a cellulose column in the same manner.

Afterwards, the sa-mRNA was concentrated, and the buffer was changed to nuclease-free H_2_O using isopropanol precipitation. To the sa-mRNA, 0.1 volume of a 3 M sodium acetate (Invitrogen, Waltham, MA, USA) solution was added with 1 volume of ice-cold isopropanol (Thermo Fisher Scientific, Waltham, MA, USA). The solution was then incubated at −20 °C for 30 min followed by centrifugation at 16,000 RCF and 4 °C for 30 min. The solution was removed from the pellet and the pellet was washed with 70% EtOH (Thermo Fisher Scientific). A final centrifugation step was executed for 10 min at 16,000 RCF, after which the EtOH could be removed. Subsequently, concentration and purity were assessed with a NanoDrop One (Thermo Fisher Scientific). Besides the concentration and purity, quality and integrity of the sa-mRNA were investigated with a bleach gel for the in vitro assays [24] and by capillary gel electrophoresis using a 5200 Fragment Analyzer (Agilent, Santa Clara, CA, USA) for the in vivo assays. The 15 nt RNA kit (DNF-471–0500, Agilent) was used with a Lonza 50,575 RNA marker (Lonza, Bornem, Belgium). The purified sa-mRNA was stored at −80 °C until further use.

#### LNP formulation and characterization

After production and purification, the sa-mRNA was formulated in two distinct LNPs, LNP 1 and LNP 2, using a NanoAssemblr Ignite (Precision Nanosystems, Vancouver, BC, Canada). For the formulation, lipid solutions with a specific composition were prepared in 100% ethanol. LNP 1 was comprised of 1,1'-((2-(4-(2-((2-(Bis(2-hydroxydodecyl)amino)ethyl)(2-hydroxydodecyl)amino)ethyl)piperazin-1-yl)ethyl)azanediyl)bis(dodecan-2-ol) (C12-200, 35 mol%, LP-04–425, CordenPharma, Brussels, Belgium), 1,2-di-(9Z-octadecenoyl)-sn-glycero-3-phosphoethanolamine (DOPE, 20 mol%, 850725P, Sigma-Aldrich), 1,2- dimyristoyl-rac-glycero-3-methoxypolyethylene glycol-2000 (DMG-PEG2000) (1.5 mol%, 880151P, Sigma-Aldrich) and cholesterol (43.5 mol%, 700100P, Sigma-Aldrich). LNP 2 consisted of C12-200 (17.5 mol%), 2-[2,2-bis[(9*Z*,12*Z*)-octadeca-9,12-dienyl]−1,3-dioxolan-4-yl]-*N*,*N*-dimethylethanamine (Dlin-KC2-DMA, 17.5 mol%, MedChemExpress, Monmouth Junction, NJ, USA), DOPE (15 mol%), DMG-PEG2000 (1.5 mol%) and cholesterol (48.5 mol%). Both LNP 1 and 2 used during the in vivo trial also contained 1 mol% DiD (1,1′-dioctadecyl-3,3,3′,3′-tetramethylindodicarbocyanine,4-chlorobenzenesulfonate salt, Thermo Fisher Scientific). In addition, the sa-mRNA solution was prepared by diluting the sa-mRNA in a 10 mM citrate buffer at pH 4.5 (Alfa Aesar, Kandel, Germany). The LNPs were prepared at a lipid/RNA (N/P) ratio of 37 and 30 for LNP 1 and 2, respectively.

To determine the concentration and encapsulation efficiency of the sa-mRNA, the Quant-it RiboGreen assay (Thermo Fisher Scientific) was used. All samples were diluted 1/20 in TE buffer and 1/200 in TE buffer containing 0.5% Triton X-100 (Acros Organics, Geel, Belgium), respectively. Next, the samples were incubated for 5 min at RT. Subsequently, 100 µL of the samples were transferred to a black 96-well plate, to which 100 µL of a ribogreen solution, diluted 1/2000 in TE buffer, was added. The plate was shaken for 5 s at 432 RPM (orbital shaking with Infinite 200 PRO M plex) and placed in the dark for 5 min at RT. Finally, the fluorescent intensity was measured using an Infinite 200 PRO M plex (Tecan, Männedorf, Switzerland) with an excitation wavelength of 485 nm and an emission wavelength of 530 nm. To determine the concentration of the sa-mRNA, ribosomal RNA was included as a standard.

The formulated nanoparticles underwent quality control assessments including size and charge analysis. Size was analyzed using a Nanosight NS300 (Malvern Panalytical) for the in vitro assays. Here, particles were diluted at a ratio of 1/200 in PBS and five 60-s videos were recorded. Subsequently, these videos were processed using the Nanoparticle Tracking Analysis software. The charge of the LNPs was measured by diluting them at a ratio of 1/20 in PBS and employing a NanoZS Zetasizer (Malvern Panalytical). The settings used were: 0.882 mPa.s for viscosity, 1.3 for refractive index and 79 for dielectric constant. For the in vivo trial, size and charge of the particles was measured using a Zetasizer Advance (Malvern Panalytical).

#### Transfections

In vitro transfections were performed with the LNP formulations to assess the gene expression under varying conditions. HeLa cells (CRM-CCL-2, ATCC, Manassas, VA, USA) were chosen as the transfection model and were cultured at standard conditions at 37 °C with 5% CO_2_. Dulbecco’s modified Eagle’s medium (DMEM) supplemented with GlutaMax, glucose and pyruvate (Gibco, Paisley, UK), 10% heat inactivated fetal bovine serum (Gibco), 2% vancomycin (Sandoz, Vilvoorde, Belgium), 1% vitamin (Gibco) and 1% streptomycin (Gibco) was used as the culture medium. Cells were seeded at 70,000 cells/well in a 24-well plate. The next day, the cells were rinsed with PBS, and 200 µL of opti-MEM (Gibco) was added to each well. Afterwards, the cells were transfected by adding 500 ng sa-mRNA encapsulated in an LNP, followed by incubation at 37 °C and 5% CO_2_. Four hours post-transfection (PT), the opti-MEM containing the LNPs was removed, and the cells were washed once with PBS before incubating with the cell culture medium. At 24 h PT, the cell culture medium was removed, and the cells were washed with PBS. Subsequently, the cells were detached using 100 µL 0.25% Trypsin EDTA (Gibco) for 5 min at 37 °C, followed by adding 100 µL cell-culture medium. Afterwards, 150 µL of the cell suspension was transferred into a black 96-well plate. Next, 10 µL D-luciferin (15 mg/mL, GoldBio, St. Louis, MO, USA) was added to the cells. The cells were incubated for 10 min at 37 °C and the luminescent signal was measured using an IVIS lumina III series (PerkinElmer, Waltham, MA, USA).

#### Resistance to spraying

The following sprayers were tested for their capability to spray the different LNPs: a PennCentury MicroSprayer model IA-1 C, the sprayer model A1, and the MADgic laryngotracheal mucosal atomization device. The LNPs were sprayed in an Eppendorf tube before being added to the cells, allowing the size of the sprayed particles to be determined using a Nanosight NS300. Equal volumes of LNPs, both before and after spraying, were used for the transfection assays as described in the “Transfections” section, corresponding to 500 ng of sa-mRNA in the non-sprayed condition.

#### sa-mRNA potency in mucus

The stability of the LNPs was tested in cervicovaginal and nasal mucus collected from pigs in the slaughterhouse. Flocked swabs (Copan, Brescia, Italy) were used to collect mucus samples from the lower reproductive tract of the pigs. Mucus samples from the nose were collected using flocked swabs immediately after slaughter, to avoid blood contamination. All swabs were transported on ice and centrifuged over a 70-micron cell strainer (VWR, Radnor, PA, USA) at 1500 RCF for 15 min and 4 °C. In the lab, the mucus samples of the different pigs were pooled, to ensure an adequate volume for testing and to minimize variations in mucin content.

A human cervicovaginal mucus simulant was prepared as described by Das Neves et al*.* [25]. To prepare the human mucus simulant, the following compounds were mixed in demineralized water: sodium chloride (0.351% W/V, VWR), potassium hydroxide (0.14%, Sigma-Aldrich), calcium hydroxide (0.0222%, Sigma-Aldrich), lactic acid (0.2%, Thermo Fisher Scientific), acetic acid (0.1%, Thermo Fisher Scientific), glycerol (0.016%, Sigma-Aldrich), urea (0.04%, Sigma-Aldrich), glucose (0.5%, Sigma-Aldrich) and porcine gastric mucin (1.5%, Sigma-Aldrich). The pH of the simulant was adjusted with hydrochloric acid to 4.2.

The pig cervicovaginal mucus, pig nasal mucus or the human simulant were mixed with an equal volume of LNP solution. The mixtures were then incubated at 37 °C for 0, 30 or 60 min. LNPs mixed with opti-MEM were included as a negative control. Subsequently, the LNP/mucus or LNP/opti-MEM solutions were added to HeLa cells, and transfections were performed as described in the “Transfections” section. The bioluminescent signal was measured 24 h PT to evaluate the LNP stability and transfection efficiency.

#### Viability of HeLa cells with mucus

As an additional control, the viability of HeLa cells incubated with mucus alone was measured to investigate whether drops in potency are linked with a reduced viability of the HeLa cells due to the mucus. HeLa cells were treated in the same manner as described above. However, mucus was now mixed with an equal volume of opti-MEM instead of LNP formulation. After 24 h, the cells were detached and 50 µL of the cell suspension was added to 50 µL HeLa medium in a clear 96 well plate. Wells containing 75 µL medium and 25 µL trypsin EDTA were also included. Next, 10 µL of WST-1 reagent (Roche, Diegem, Belgium) was added to the cell suspension. The cells were incubated for 30 min at 37 °C and 5% CO2 and the absorbance were measured with a Tecan plate reader at 450 nm and a reference wavelength of 620 nm after shaking for 1 min. The viability was calculated compared to the average absorbance of the untreated HeLa cells after subtraction of the medium background signal.

#### Screening of lipid nanoparticles (LNPs) in pigs

The Ethical Committee of the Faculty of Veterinary Sciences of Ghent University approved the in vivo trial (EC2021-022). Two trials were conducted each involving 21 female pigs (*Sus scrofa domesticus*), aged six weeks, and sourced from a local farm. The pigs were housed in stables with unrestricted access to water and food. Upon arrival, they received an oral administration of Colistin (Colivet Quick Pump, Prodivet Pharmaceuticals, Eynatten, Belgium) to prevent bacterial gut infections due to stress during the transport. Twenty-four hours before administration of the LNPs, food and water were withheld. For LNP administration, pigs were sedated by intramuscular injection of a mixture of tiletamine hydrochloride and zolazepam hydrochloride (Zoletil^®^100, Virbac Animal Health, Louvain La Neuve, Belgium) and 2% Xylazine-M (VMD, Arendonk, Belgium). Next, the pigs were treated both intravaginally and intranasally with 15 µg sa-mRNA::*fluc* encapsulated in DiD-labelled LNP 1 or LNP 2 in 250 µL PBS, either through injection or spraying. The injection was performed by dividing the dose between two injection sites using a 29 G insulin syringe (KDM Medical GMBH medical products, Berlin Germany). The custom sprayer A1 design was used for the mucosal administration. For the intravaginal administration, the tip of the sprayer was inserted into the vulva and gently advanced until it reached a depth of approximately two cm inside the vagina. For the intranasal administration, the sprayer tip was inserted to a similar depth in the nasal cavity. After administration, the head or the pelvis of the animal was slightly elevated to prevent leakage of the formulation from the administration site. For both the injection and the spray, the left nasal cavity of the pig was treated, while the right cavity was left untreated.

The pigs were euthanized 1-, 2- or 4 days post-treatment by an intravenous administration of an overdose of pentobarbital (Euthanimal 70 mg/kg; Nembutal^®^, Ceva Santé Animale, Maassluis, the Netherlands). Following exsanguination, the left nasal conchae and the lower reproductive tract were isolated with their respective local draining lymph nodes. Both inguinal lymph nodes were collected for the genital mucosa, while the left mandibular and left retropharyngeal lymph nodes were collected for the nasal mucosa. The tissues and lymph nodes were transported on ice in Hank’s Balanced Salt Solution (HBSS, Thermo Fisher Scientific) before measuring the signal using an IVIS lumina III series (Perkin Elmer). First, the fluorescent signal was measured to check for LNP uptake. Subsequently, 15 mL of a 1.5 mg/mL luciferin (GoldBio) in PBS solution was applied on top of the tissues to measure the bioluminescent signal. The signal was measured every 2 min using the following settings: automatic exposure time and binning set at 16. Regions of interest (ROIs) were quantified for each tissue at the average time when the respective tissue reached its peak signal. Spectral unmixing was conducted to subtract the background signal from the fluorescent signal.

### Statistical analysis

Figures and statistical analyses were performed in GraphPad Prism (version 10.3.1). Normality and equality of variances were assessed. ANOVA was used to compare the different groups with their respective control measurement. For the viability assay, a Welch ANOVA was performed. As a post-hoc test, Tukey’s, Dunnett’s, or Sidak method for multiple comparison were performed where applicable. In case normality was not achieved, the non-parametric Kruskal–Wallis test was performed. Statistical significance is indicated in the figures as follows: P < 0.05 *, P < 0.01 ** and P < 0.001 ***.

## Results

### Droplet size comparison reveals notable disparities among three sprayers designed for mucosal applications

**Fig. 1:**
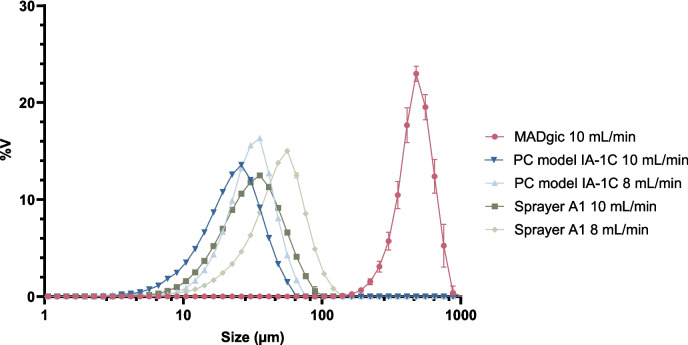
Droplet size distributions generated by the three sprayers. Water was sprayed using an HPLC pump at two different flow rates. The resulting droplets size was measured as three independent repeated measurements. The X-axis represents the droplet size in µm, while the Y-axis represents the volume-weighted distribution (%V). Error bars indicate the standard deviation (SD) between the measurements

Mucosal spraying is the only applicable administration method for mucosal administration. This method enables vaccine deposition over a larger surface, potentially mimicking a natural infection. The aerosol particle size and the sprayer are critical factors influencing mucosal drug deposition. For instance, droplets larger than 20 µm preferentially deposit in the nasal cavity, while droplets smaller than 10 µm reach the respiratory tract [26]. Of these two deposition sites, only nasal vaccination can generate an immune response at the infection site of STIs. On the other hand, direct intravaginal application was also considered as a control to directly establish immune responses at the site of infection and since women are disproportionally affected. For this route, a concise administration volume is needed to avoid spill-out. Therefore, we selected three jet sprayers that meet these requirements, *i.e.* the MADgic atomization device, the PennCentury model IA-1 C and model A1. The droplet size distributions of these sprayers were measured by laser diffraction (Fig. 1).

The PennCentury model IA-1 C generated the smallest droplets, with a volume-weighted mean droplet diameter of 22.4 ± 0.1 µm and 27.3 ± 0.1 µm at 10 mL/min and 8 mL/min, respectively. Model A1 generated a volume-weighted mean droplet size of 31.3 ± 0.6 µm and 46.7 ± 0.4 µm at the respective flow rates. Both sprayers had a similar droplet size distribution, as illustrated in Fig. 1. In contrast, the MADgic produced larger droplets with a volume-weighted mean size of 442 ± 17 µm at 10 mL/min. It was impossible to measure the mean droplet size at 8 mL/min. During multiple attempts, we visually observed the droplets to be larger, hindering their passage through the laser using the same setup. Thus, the PennCentury model IA-1 C and the novel sprayer model A1 generated similar droplet size distributions, while the MADgic sprayer produced droplets with a bigger size at the same flow rate.

### sa-mRNA maintains its potency and the LNPs retain their physicochemical properties after spraying

**Fig. 2:**
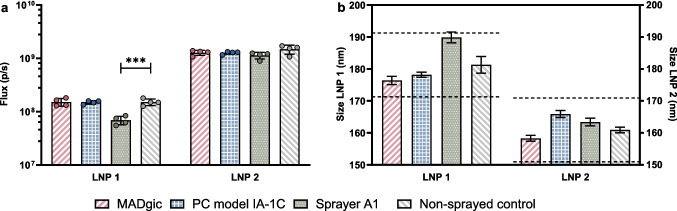
The bioluminescent signal obtained after transfection with sa-mRNA encapsulated in LNP 1 or LNP 2 (**a**) and their particle size (**b**) before and after spraying. a. Sprayed and non-sprayed particles were used to transfect HeLa cells. The Y-axis represents the bioluminescent signal measured in photons per second (p/s). The mean signal (bar) and the individual measurements (n = 4) are given with the SD (error bars). Significance is indicated by P < 0.001***. (**b**). The LNP size of the particles used for transfection was measured as three repeated measures. The Y-axis gives the particle size with a range indication of 10 nm around the non-sprayed control. The bars depict the mean droplet size, while the error bars give the SD

Next, we investigated whether the potency of the sa-mRNA LNP vaccine was influenced by spraying the particles using the three sprayers described above. The forces exerted on the particles during spraying can disrupt the LNP structure and damage the encapsulated sa-mRNA [27]. Two distinct LNP formulations were investigated: LNP 1 and LNP 2. A tenfold increase in expression during in vitro transfections was observed when using LNP 2 compared to LNP 1 (Fig. 2.a). The bleach gel to check the sa-mRNA quality and the size and zeta potential of the non-sprayed particles used during the in vitro study are shown in the supplementary figures (Fig. S1 and S2 respectively). After spraying, the size of the particles was remeasured (Fig. 2.b). Next, HeLa cells were transfected with these sprayed particles to evaluate sa-mRNA potency after spraying (Fig. 2.a). A similar potency and particle size before and after spraying indicates that this process does not affect LNP performance and its physicochemical characteristics.

For sa-mRNA formulated in LNP 1, all conditions resulted in a signal intensity after transfection of 10^8^ photons/second (p/s) except for model A1. The signal intensity of LNP 1 sprayed with model A1 (6.9 × 10^7^ ± 1.3 × 10^7^ p/s) was significantly lower (P < 0.001), compared to the non-sprayed control (1.5 × 10^8^ ± 2.2 × 10^7^ p/s) (Fig. 2.a). Despite the decrease of 55% in expression, signals were still 100 times higher compared to the background signal (order of 10^5^ p/s). Transfections of sa-mRNA formulated in LNP 2 resulted in a higher expression of 10^9^ p/s across all conditions compared to LNP 1 (10^8^ p/s). Additionally, all sprayed particles had sizes within 10 nm of their respective non-sprayed controls. Altogether, we concluded that spraying did not affect the size of the LNPs or the potency of the sa-mRNA within them. Based on the results of the spraying experiments, we decided to use sprayer A1 in the upcoming in vivo experiment for the following reasons. The MADgic produced larger droplets than both the PennCentury and custom sprayer A1, which increases risk for leakage from the nasal cavity. Since the custom sprayer A1 is based on the PennCentury microsprayers design, we included the discontinued PennCentury microsprayer as a control for the A1 sprayer. Since the overall performance of the A1 sprayer was still acceptable, we decided to continue with the newly produced microsprayer.

### Potency of sa-mRNA encapsulated in LNPs is maintained when incubated with porcine mucus in vitro

**Fig. 3:**
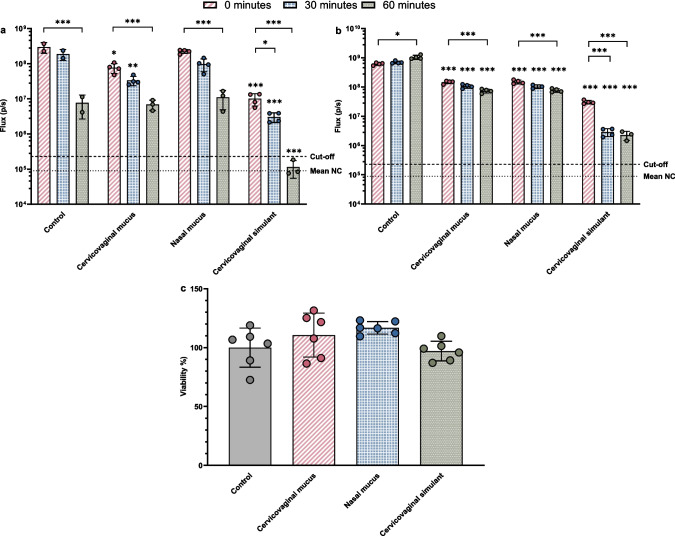
Potency of the sa-mRNA reporter construct in LNP 1 (**a**) or LNP 2 (**b**) in cervicovaginal and nasal pig mucus and a human mucus simulant, and the viability of HeLa cells incubated with mucus (**c**). The bioluminescent signal is depicted on the Y-axis as the flux (p/s). The mean signal (bar) and the individual measurements are given with the SD (error bars). The dashed line indicates the mean negative control (NC) signal. The cut-off value, represented by a 2nd dashed line, is calculated by adding two times the standard deviation to the mean negative control signal. Significance is denoted by P < 0.05 *, P < 0.01 ** and P < 0.001*** on top of the bar to compare with the control measurement at the same time point or within the group between the time points. c. Viability of HeLa cells (Y-axis) incubated with the different mucus conditions (X-axis) as a percentage in comparison to the untreated control cells. The mean viability (bar) and the individual measurements are given with the SD (error bars). For each condition, six biological replicates were included. No significant differences were found compared to the control

When sprayed onto a mucosal surface, LNP particles first enter the mucus layer, which they must traverse to reach the underlying cell layer. LNPs need to retain their potency and stability within the complex mucus layer to ensure effective diffusion and delivery of the sa-mRNA to the cells below. Therefore, we tested whether mixing the LNPs with porcine cervicovaginal mucus, nasal mucus or a human mucus simulant might influence these characteristics. The used mucus types represent potential administration sites for a future sa-mRNA-based STI vaccine in the pig model and for human applications. Unfortunately, we could not measure the physicochemical characteristics of the LNPs incubated with mucus. As mucus is a complex biological mixture containing many different proteins, it was impossible to achieve good measurements using dynamic light scattering methods. The potency of the LNPs was assessed by transfecting HeLa cells with LNPs pre-incubated with the different mucus types (Fig. 3.a and b). As a control, the viability of HeLa cells incubated with the different mucus types was also examined (Fig. 3.c).

For LNP 1 pre-incubated with opti-MEM (control), luciferase expression significantly dropped over time from 3.0 × 10^8^ ± 1.0 × 10^8^ p/s at 0 min of incubation at 37 °C to 7.7 × 10^6^ ± 5.1 × 10^6^ p/s at 60 min (P < 0.001) (Fig. 3.a). Incubating the LNP 1 formulations in mucus also led to an approximately 10- to 20-fold decrease in expression over time for the porcine mucus conditions and a complete loss of luciferase expression when incubated in the human vaginal simulant (P < 0.001).

When comparing with the positive control measurements at each time point, the LNP 1 formulations incubated in porcine nasal mucus showed no significant differences (Fig. 3.a). In porcine vaginal mucus, the LNP 1 formulation showed a significant difference in expression compared to the positive control at 0 (P < 0.01) and 30 min (P < 0.001), but not at 60 min. Although expression dropped significantly compared to the positive control, the sa-mRNA potency is still retained achieving sufficient expression levels above the cut-off. In the human mucus simulant, LNP 1 formulations showed a steeper decrease in expression compared to the positive control (P < 0.001). At 0 and 30 min, expression dropped 30-fold to 1.0 × 10^7^ ± 3.9 × 10^6^ p/s and 100-fold to 3.1 × 10^6^ ± 9.4 × 10^5^ p/s, respectively. At 60 min, the bioluminescent signal dropped below the cut-off for expression, losing its potency.

Unlike LNP 1, no bioluminescent values were detected below the cut-off for any LNP 2 sample, indicating that all conditions showed expression (Fig. 3.b). However, changes in expression over time were also seen for LNP 2. The expression of LNP 2 formulations incubated in opti-MEM slightly increased over time, from 6.2 × 10^8^ ± 6.0 × 10^7^ p/s at 0 min to 1.1 × 10^9^ ± 1.7 × 10^8^ p/s at 60 min (P < 0.05). On the other hand, LNP 2 mixed with either one of the three mucus types resulted in a significant decrease in luciferase expression over time.

When comparing the mucus incubated conditions with the LNP 2 control measurement, the signal decreased from 6.2 × 10^8^ ± 6.0 × 10^7^ p/s at 0 min to 1.5 × 10^8^ ± 1.9 × 10^7^ p/s for the formulations incubated in porcine cervicovaginal and nasal mucus and to 3.1 × 10^7^ ± 4.2 × 10^6^ p/s for the human cervicovaginal simulant (P < 0.001) (Fig. 3.b). At 30 and 60 min, the signal continued to gradually decrease, reaching 7.4 × 10^7^ ± 1.1 × 10^7^ p/s in pig vaginal mucus, 7.7* × 10^7^ ± 1.1 × 10^7^ p/s in porcine nasal mucus, and to 2.3 × 10^6^ ± 7.7 × 10^5^ p/s in the human simulant (P < 0.001). However, the potency of LNP 2 dropped the steepest when incubated in the human mucus simulant, with a 20-, 240- and 460-fold decrease in luciferase expression at 0, 30 and 60 min, respectively.

Furthermore, the viability of the HeLa cells was retained after incubation with the different mucus conditions (Fig. 3.c). No significant differences in viability were seen compared to the untreated control cells. Differences in bioluminescent signal are thus caused by interactions between the mucus and the LNP. Therefore, based on these measurements, the LNPs largely retained their potency in cervicovaginal and nasal pig mucus, while the potency dropped more severely when the particles were incubated in a human mucus-simulant.

### LNP encapsulated sa-mRNA vaccines are applicable in vivo using injection and spraying

**Fig. 4:**
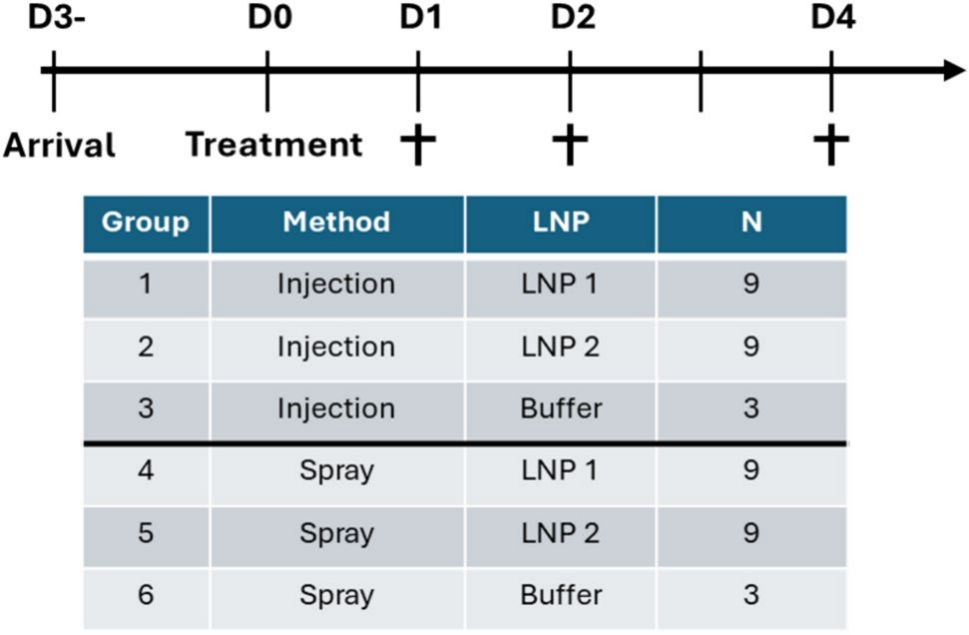
Timeline of the in vivo trial and group allocation. The sa-mRNA encoding firefly luciferase was encapsulated in fluorescently labelled LNP 1 or LNP 2. The particles were administered through intranasal and intravaginal injection or spraying at a dose of 15 µg in 250 µL. At 1-, 2- and 3- days post-treatment, the treated mucosa and local draining lymph nodes were collected for ex vivo visualization using an IVIS lumina III

In vitro evaluation of the two sa-mRNA LNP-reporter vaccines demonstrated that the potency of the sa-mRNA is retained after spraying and when incubated in pig mucus. Next, we wanted to ensure their applicability for in vivo mucosal administration using pigs as a model organism. The two reporter vaccine constructs were administered through mucosal injection (control) and spraying during two separate in vivo studies using 21 pigs per administration method. The quality of the sa-mRNA was checked with a fragment analyzer (Fig. S3). A comparison of the physicochemical properties of the LNPs and the potency of the sa-mRNA revealed no major differences between the different batches of LNPs (Fig. S4 and S5). The set-up of the in vivo trials and the different groups can be found in Fig. 4, together with the graphical abstract. An overview of the plate set-up and representative images taken from the IVIS are included in the Appendix (Fig. S6). All animal samples were analyzed within one-hour post-exsanguination.

**Fig. 5:**
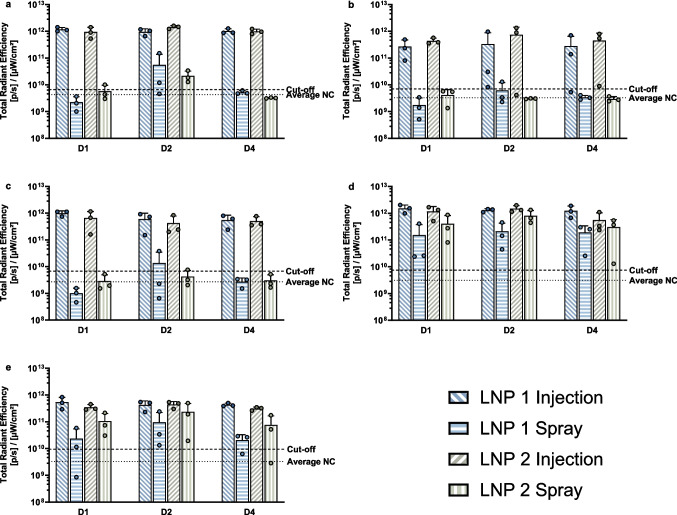
Fluorescent signal measured in the mucosal tissues and their local draining lymph nodes 1-, 2- and 4-days post-administration. Fluorescent signal was measured at the nasal mucosa (**a**), the mandibular lymph node (**b**), the retropharyngeal lymph node (**c**), the vaginal mucosa (**d**) and the inguinal lymph node (**e**). The fluorescent signal can be found on the Y-axis as total radiant efficiency ([p/s]/[µW/cm2]). The mean values per group and the individual values (3 replicates per group) are depicted on the graph. The mean signal of the negative control (NC) is presented by a dashed line, and a threshold for the positive signal is established by adding twice the standard deviation to this mean (2.^nd^ dashed line)

In the nasal mucosa of all animals receiving the LNPs through injection, a fluorescent signal was detected with values ranging from 5.5 × 10^11^ to 1.6 × 10^12^ [p/s]/[µW/cm^2^] (Fig. 5.a). A fluorescent signal was also observed in the local draining lymph nodes, although with more variation and a 50% lower intensity compared to the nasal mucosa (Fig. 5.b and c). While for all animals a fluorescent signal was present in the retropharyngeal lymph nodes (Fig. 5.c), not all animals showed signals in the mandibular lymph node (Fig. 5.b). The mandibular lymph node of one of the three animals receiving LNP 1 and sacrificed on day 4, and one of the three animals receiving LNP 2 and sacrificed on day 2, showed no fluorescent signal above the cut-off.

In contrast to injection, spraying of the LNPs resulted in a limited fluorescent signal in the nasal mucosa (Fig. 5.a) and its draining lymph nodes (Fig. 5.b and c). Of the three animals receiving LNP 2 via spraying and sacrificed on day 1, only one showed a fluorescent signal above the cut-off (6.6 × 10^11^ [p/s]/[µW/cm^2^]). The fluorescent signal increased above the cut-off for the animals sacrificed on day 2 after administration. Here, the signal was below the cut-off for only one animal receiving LNP 1. Four days after administration through spraying, the fluorescent signal could no longer be observed for either LNP formulations. On day 2, the retropharyngeal lymph node of one pig receiving the sprayed LNP 1 formulation and one pig receiving the sprayed LNP 2 formulation were identified as a responder (Fig. 5.c). Additionally, the mandibular lymph node of one animal receiving LNP 1 through spraying showed a signal above the cut-off on day 2 (Fig. 5.b). Overall, animals showed no prominent differences in signal intensity when treated intranasally with LNP 1 or LNP 2.

In the vaginal mucosa (Fig. 5.d), spraying of the LNPs did result in the detection of a local fluorescent signal. However, compared to injection as an administration method, spraying showed a higher inter-animal variability and an approximately tenfold lower signal. An average fluorescent signal of 1.2 × 10^12^ ± 5.3 × 10^11^ [p/s]/[µW/cm^2^] was observed in animals receiving injections, compared to 3.5 × 10^11^ ± 3.5 × 10^11^ for administration through spraying. The inguinal lymph nodes of all animals receiving injections showed signals above the cut-off (9.5 × 10^11^ [p/s]/[µW/cm^2^], Fig. 5.e). For the groups treated with sprayed particles, the lymph nodes of all the animals exhibited a true fluorescent signal above the cut-off, except for one of the three animals receiving LNP 1 on day 1 post spraying and one of the three receiving LNP 1 and LNP 2 on day 4 post spraying. A threefold higher signal was observed in the vaginal mucosa and draining lymph nodes of animals receiving a sprayed LNP 2 formulation (5.1 × 10^11^ ± 4.1 × 10^11^ and 1.4 × 10^11^ ± 1.6 × 10^11^ [p/s]/[µW/cm^2^], respectively), compared to those receiving sprayed LNP 1 (1.9 × 10^11^ ± 1.7 × 10^11^ and 4.7 × 10^10^ ± 7.5 × 10^10^ [p/s]/[µW/cm^2^], respectively). The fluorescent signal intensity at the vaginal mucosa (10^12^ [p/s]/[µW/cm^2^]) and the inguinal lymph node (10^11^) were comparable between the two LNP formulations when administered through injection.

**Fig. 6:**
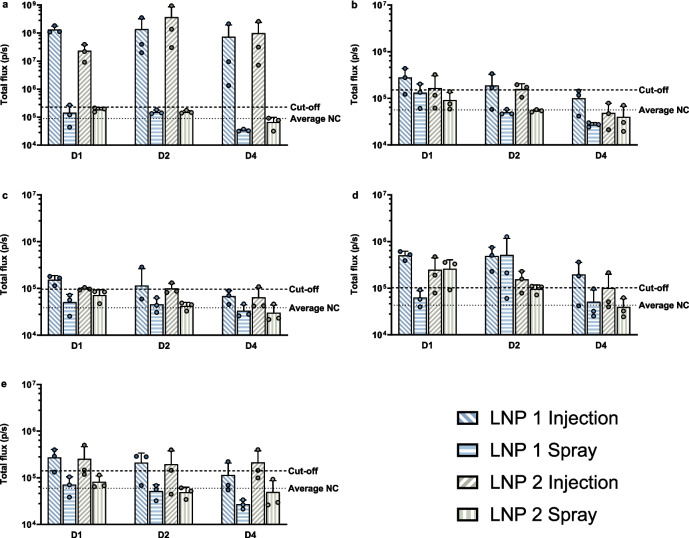
Bioluminescent signal (Y-axis, p/s) measured in the mucosal tissues and their local draining lymph nodes 1-, 2- and 4-days post-administration (X-axis). The bioluminescent signal was measured at the nasal mucosa (**a**), the mandibular lymph node (**b**), the retropharyngeal lymph node (**c**), the vaginal mucosa (**d**) and the inguinal lymph node (**e**). The mean values per group and the individual values (3 replicates/group) are depicted on the graph. The mean signal of the negative control (NC) is presented as a dashed line, and a threshold for the positive signal is established by adding twice the standard deviation to this mean (dashed line)

Bioluminescent signals were measured to analyze the expression of the sa-mRNA. No bioluminescent signal was detected in the nasal mucosa of animals receiving LNPs through spraying, except for one animal that received LNP 1 and was sacrificed on day 1 (2.7 × 10^5^ p/s, Fig. 6.a). Similarly, the mandibular (Fig. 6.b) and retropharyngeal lymph nodes (Fig. 6.c) of the animals receiving LNPs through spraying showed no bioluminescent signal, except in one animal, that received LNP 1 and was sacrificed on day 1, which showed bioluminescence in the mandibular lymph node (2.0 × 10^5^ p/s with cut-off 1.5 × 10^5^). In contrast, when LNPs were administered by injection, a bioluminescent signal was observed in both the nasal mucosa and the local draining lymph nodes. In the nasal mucosa, there was a large variation in bio-expression, with signals ranging from 1.3 × 10^6^ p/s in the lowest responder to 9.4 × 10^8^ p/s in the highest. Generally, most signals had intensities between 10^7^ p/s and 10^8^ p/s. The mandibular lymph node of animals injected with LNP 1 or LNP 2 showed a bioluminescence signal when sacrificed on day 1 (2/3 for LNP 1 and 1/3 for LNP 2) and day 2 (2/3 for both). Bioluminescence was only seen in the retropharyngeal lymph node of animals receiving the LNPs through injection (Fig. 6.c). On day 1, all animals receiving LNP 1 and two animals receiving LNP 2 displayed a signal. This changed to only one of the three animals for both LNPs on day 2 and one animal on day 4 for LNP 2. The highest lymph node signal only exceeded the cut-off by a threefold increase (maximum of 4.4 × 10^5^ p/s), meaning that overall low signals were obtained at the lymph nodes.

At the vaginal mucosa (Fig. 6.d), a bioluminescent signal was detected in both the injection and spray groups. However, in both cases, the signal was weak (maximum signal of 10^6^ p/s) and highly variable. On days 1 and 4 post-administration, pigs receiving LNP 1 through spraying did not express a bioluminescent signal, while two of the three pigs showed a signal on day 2. In contrast, pigs receiving LNP 1 through injection displayed a signal in all animals on all days, except for one pig sacrificed on day 4. Spraying of LNP 2 resulted in expression in two of the three pigs sacrificed on days 1 and 2, but no expression on day 4. Pigs receiving injections had a similar pattern, except for day 4, where one of the three pigs showed expression. In general, signals were also two-fold lower for LNP 2 compared to LNP 1. The inguinal lymph nodes of animals receiving the LNPs via spraying did not show any bioluminescent signal (Fig. 6.e). For animals treated through injection, a signal was present for two of the three animals for each LNP on all days, except for LNP 1 on day 4 (1/3). The highest signal was nearly four times higher than the cut-off (5.0 × 10^5^ p/s).

To conclude, both fluorescent and bioluminescent signals were detected in the nasal and vaginal mucosa, as well as their draining lymph nodes, following injection of LNP 1 or LNP 2. In contrast, spraying of the LNPs did not show any fluorescent or bioluminescent signals in the nasal mucosa or its draining lymph nodes. Although signals were detected in the vaginal mucosa following spraying, they were weaker and more variable compared to those observed in animals receiving the LNPs through injection.

## Discussion

Sexually transmitted infections (STIs) remain a significant global public health challenge affecting millions of individuals each year. Despite advances in medicine and public health interventions, the burden of STIs continues to rise [1]. One of the most pressing concerns in STI prevention is the lack of effective vaccines. It is theorized that local mucosal immune responses and the generation of tissue-resident memory cells through mucosal vaccination are necessary to achieve protection. However, mucosal vaccination is impeded by the inherent barriers of these sites, such as the mucus layer, tight intracellular junctions and the tolerogenic barrier [9, 10].

We hypothesized that combining the self-adjuvating effect of sa-mRNA with the immunogenic properties of LNP carriers might provide a superior platform to overcome the tolerogenic barrier at mucosal surfaces without the need for additional mucosal adjuvants. However, the mucus barrier might limit delivery of the LNP formulations. We aimed to demonstrate that LNP-sa-mRNA formulations can be administered mucosally and can thus potentially be used in the development of prophylactic vaccines to protect against STIs.

However, up till now, research describing mucosal sa-mRNA vaccination is limited and contradictory. For example, Anderluzzi et al*.* [16] reported weak immune responses and rapid clearance of sa-mRNA expression, whereas Jennewein et al*.* [15] observed strong systemic and mucosal immune responses following intranasal sa-mRNA vaccination. Additionally, Anderluzzi et al*.* [16] showed different levels of expression depending on the specific LNP formulation. These inconsistencies might be explained by the mucus barrier which interferes with the uptake of certain nanoparticles depending on its characteristics.

To address these inconsistencies, our initial goal was to ensure that mucosal uptake and expression of sa-mRNA were achievable using pigs as a larger animal model before progressing to an immunization study. We investigated two different sa-mRNA containing LNPs, as well as different administration methods and routes. Pigs were used to investigate mucosal delivery as their nasal and reproductive anatomy is more comparable to humans than that of mice [19, 28]. The LNP formulations were delivered at the mucosal sites using spraying as an attractive needle-free delivery method for these sensitive tissues. However, recognizing that the mucus barrier at the mucosa can entrap and remove foreign particles, injection was included as a control to bypass this barrier. The LNPs were administered either intranasally or intravaginally. Intranasal vaccination can elicit immune responses in the reproductive tract through the common mucosal immune system [6]. Direct intravaginal application was considered because women are disproportionally affected by STIs. However, intravaginal vaccination for humans faces significant ethical and scientifical hurdles. Therefore, it is more considered for the pre-clinical phase as a control group for the direct establishment of immune responses at the site of infection and as an administration route for potential porcine applications.

In the nasal mucosa and its local draining lymph nodes, a fluorescent and bioluminescent signal was only observed in the animals receiving the LNPs through injection. This demonstrates that expression of the sa-mRNA is possible in the nasal mucosa, consistent with the observations of Anderluzzi et al*.* [16]. They reported a bioluminescent signal in mice following intranasal inhalation of sa-mRNA encapsulated in four distinct lipid-based nanoparticles. Contrarily, we did not observe any sa-mRNA expression when administering intranasally in pigs through spraying. While a fluorescent signal was detected in some animals, it was much lower than the signal observed for the injection group. Since we demonstrated during the in vitro stage that the size and transfection efficiency of the particles were unaffected by the spraying process, it is unlikely that the particles were damaged by the spray force. The stability of free and encapsulated mRNA after spraying has also been demonstrated by other studies [29, 30]. As only a limited fluorescent signal was observed in some animals, the lack of protein expression is most likely due to insufficient delivery of the LNPs at the nasal mucosa.

A first potential explanation for the delivery issues lies in the deposition of droplets in the nasal cavity. Droplet size greatly influences deposition patterns in the upper and lower respiratory tract. According to Schroeter et al*.* [26], particles larger than 20 µm in size tend to deposit more in the anterior part of the nasal cavity compared to 10 µm particles. Therefore, during the in vitro stage, droplet size measurements of different sprayers were performed to ensure the correct particle size for nasal deposition. None of the tested sprayers produced droplets smaller than 10 µm. Indeed, the smallest droplets were generated by the PennCentury model IA-1 C and the custom sprayer A1. For the in vivo trial, we continued with the custom-made sprayer based on the PennCentury model IA-1 C design. The sprayer produced droplets that were small enough to avoid flow-out from the nasal cavity, yet large enough to ensure deposition in the anterior nasal cavity (< 20 µm). However, to definitively exclude posterior deposition of the particles, a larger section of the respiratory tract should be collected for visualization during future trials. Furthermore, these droplet sizes have been determined by spraying a water solution. However, since the physico-chemical properties of an LNP solution are different than those of water, the droplet size might also be affected. Therefore, it would be interesting to further validate the droplet size with a solution with more comparable characteristics.

A second and more likely explanation is that the particles were lost in the mucus layer. The loss of particles is unlikely due to stability issues in nasal pig mucus, as we demonstrated no decrease to background levels in potency of the particles incubated with porcine nasal mucus. However, a key limitation of the mucus stability assay is that it did not account for diffusion speed through the mucus. In the nasal cavity, particles have approximately 20–30 min to traverse the 10–15 µm thick mucus layer before being cleared by mucociliary clearance [31]. The mucus diffusion rates of the particles tested during the in vivo trial were likely insufficient to transport the LNP through the mucus layer. In contrast, intranasal injection of the same nanoparticles bypassed the mucus barrier, resulting in both fluorescent (LNP delivery) and bioluminescent (RNA expression) signals.

Additionally, intravaginal spraying of the particles also resulted in a fluorescent and bioluminescent signal. As the mucus turnover rate at the vaginal mucosa is approximately 24 h and thus longer than the nasal mucosa [32], the mucus diffusion speeds of the particles may be fast enough for the vaginal but not the nasal mucosa. Therefore, it would be interesting to determine the mucus diffusion rate of these particles, which can be done with one of the techniques reviewed in Lock et al. [17]. An attempt was made to establish the mucin diffusion rates with a Nanosight NS300 but components in the mucus interfered with particle tracking.

Also, the effect of the animal model and the LNP characteristics affecting mucosal deposition are two intriguing areas for further investigation. Our findings contradict those of Anderluzzi et al*.* [16] who reported local expression of sa-mRNA after intranasal administration of various lipid-based carriers. Similarly, Ongun et al. [33] demonstrated nasal expression after intranasal administration (non-sprayed droplets) of non-replicating mRNA in a C12-200-based LNP, comparable to LNP 1. However, both studies used mice as an intranasal delivery model, which is not the most representative model for this purpose [19]. As the nasal cavity of a mouse is too small to insert a nasal spray, spraying of the particles in the nasal cavity cannot be tested. In contrast, the pig model allows direct spraying in the nasal cavity while also being immunologically and anatomically more comparable to humans than mice [19]. Therefore, it would also be interesting to examine the particles used by Anderluzzi et al. [16] in pigs to see if the observed differences originate from the animal model, the administration method, or the carrier.

Additionally, there is currently no consensus on which LNP characteristics influence mucosal delivery. Regarding the two different LNP formulations in our study, no major differences were observed between LNP 1 and 2. During in vitro experiments, we observed a tenfold increase in bioluminescent signal with LNP 2 compared to LNP 1, which was not observed in vivo. Both LNP particles showed a similar potency upon spraying or incubation with different mucus types in vitro. The addition of a second ionizable lipid, Dlin-KC2-DMA, to the LNP formulation together with C12-200 did not affect the mucosal delivery. To our knowledge, the effect of a second ionizable lipid in the LNP formulation on the mucoadhesive or mucopenetrative properties of the LNP has not yet been described. This might explain the comparable in vivo outcomes observed for both LNP particles [18]. When comparing our particles with those of Anderluzzi et al. [16] and other, more distinct, lipid carriers could identify key features of mucosal lipid-based carriers, aiding in the development of new mucosal therapeutics and vaccines.

Interestingly, we demonstrated that mucosal injection in the vaginal mucosa resulted in a weaker bioluminescent signal (10^5^–10^6^ p/s) compared to the nasal mucosa (10^7^–10^8^ p/s). This disparity could be due to differences in the epithelial structure. The vaginal epithelium is a multilayered stratified squamous epithelium, similar to the epidermis of the skin. Its outermost layer, the *stratum corneum*, consists of a cornified cell layer. These cells lost their ability for protein and mRNA synthesis during cornification. Therefore, to achieve expression, the particles need to reach the underlying tissue starting from the suprabasal layer [34]. Additionally, emitted light might be partially absorbed by the *stratum corneum*, further reducing the detectable signal [35]. In contrast, the nasal epithelium is composed of a pseudostratified columnar ciliated epithelium [31]. Therefore, expression can occur at a more superficial cell layer compared to the vaginal mucosa and less signal can get lost through absorption.

An alternative would be to homogenize the tissues and measure the bioluminescent signal. This would reduce the absorbance of light and increase luciferin uptake. However, this would require extensive disruption of the mucosal tissues, especially of the cartilage containing nasal tissue. Additionally, the proteases in the mucus layer might interfere with the readout. Therefore, additional optimization would be needed to confirm this method as an alternative for ex vivo visualization with an IVIS.

Leyman et al*.* [36] reported a comparable signal intensity from pig skin biopsies after intradermal delivery of 20 µg sa-mRNA encoding for luciferase through electroporation. A comparable intensity was also found in sheep when 750 µg of naked mRNA was administered through spraying at the distal part of the vagina [37]. We achieved a similar signal with a significant lower dose of 15 µg sa-mRNA. It would be interesting to see if an increased dose of sa-mRNA could further increase the observed signal. Nevertheless, it is crucial to balance the increasing dose with platform optimizations to achieve the desired bioluminescent signal.

However, Lindsay et al*.* [37] performed their experiments with naked mRNA instead of encapsulated sa-mRNA. Their dose of 750 µg naked mRNA can probably be reduced when encapsulating the mRNA inside an LNP. Therefore, a more detailed comparison between sa-mRNA and mRNA, both encapsulated in similar LNPs for mucosal administration, would be valuable. Such experiments would allow for a more comprehensive assessment of the advantages and disadvantages, and dose differences of each platform.

The expression of both sa-mRNA LNP formulations in the vaginal mucosa disappeared by day 4 in all animals after spraying and in some animals after injection. This can be explained by the turnover rate of vaginal epithelial cells. Due to an increase in the integrity of epithelial junctions when traversing the vaginal epithelium, the sprayed particles will probably transfect cells in the suprabasal layer located beneath the *stratum corneum* [38]. The epithelium of the vagina consists of 28 cell layers, and it takes approximately 96 h for a new keratinocyte to move from the basal layer to the outermost layer in the *stratum corneum*. Therefore, the epithelium of the vaginal mucosa is entirely renewed within four days, which might explain the loss of expression at day 4 [34]. By contrast, LNPs delivered by injection can reach the *lamina propria*, resulting in residual expression at day 4. This suggests that the particle delivery method and epithelial turnover rates are critical factors influencing the duration and intensity of sa-mRNA expression in epithelial layers.

While the bioluminescent signal reduced over time, the fluorescent signal of the LNPs was still present 4 days after administration. A similar scenario was described by Lindsay et al*.* [37], where 750 µg or 250 µg naked mRNA was sprayed in the vagina and cervix of sheep or rhesus macaques respectively. The expression of a neutralizing HIV antibody (PGT121) coupled to NanoLuc; either in secreted or membrane-tethered form, was investigated. They could detect the anchored antibody for up to 1 month in the reproductive tract of sheep, while the expression of the secreted nanobody was significantly reduced 14 days after administration. However, in the reproductive tract of the rhesus macaques, a marked decrease in mRNA presence after 72 h was observed. This large discrepancy in detection time of the mRNA and the antigen was explained by the authors through initial detachment of the GPI anchored antigen and retethering at a lower site in the reproductive tract. By doing so the antigen was not removed during the replacement of the vaginal epithelial layer every 4 days, which was not the case for the mRNA. In our study, the fluorescent dye can also be transferred to other cells, retaining their presence in the reproductive tract for a longer period than the sa-mRNA or the luciferase inside the epithelial cells.

Importantly, we observed bio-expression in the inguinal lymph node only when the LNPs were administered through injection and not by spraying. However, a fluorescent signal was observed for both administration methods. Therefore, it is likely that only the DiD label could transit to the lymph node when the formulations were administered using spraying. Contrarily to our results, Lindsay et al*.* [37] demonstrated drainage of radioactive labelled naked mRNA to the lymph node after intravaginal spraying in non-human primates. The difference in lymph node drainage should be further investigated as this can influence the immunogenicity of a potential vaginal vaccine. It might be interesting to look on a cell level using the method of Lindsay et al*.* [37] or a flow cytometric approach to clarify drainage of LNP formulations to the lymph node after intravaginal administration.

Another important parameter to investigate before advancing these LNPs is the general tolerability of these formulations after mucosal administration. To investigate this, immunohistochemical staining of tissue sections should be made after mucosal administration of the LNP formulation to identify any signs of acute inflammation or necrosis. However, since we wanted to detect expression of the sa-mRNA over the entire mucosal tissue, these two assays could not be combined. Nevertheless, investigation of the tolerability should be combined with an eventual immunogenicity study or toxicology study.

Furthermore, we found a discrepancy in size of our particles when comparing the Nanosight measurements, used during the in vitro evaluation, and the Zetasizer, used during the in vivo evaluation. Using the Zetasizer, the size of LNP 1 was approximately 90 nm, while LNP 2 measured around 80 nm. However, when characterized with the Nanosight, the mean sizes were 132.3 nm for LNP 1 and 127.7 nm for LNP 2. The Zetasizer measures intensity-weighted distributions, whereas the Nanosight provides number-weighted distributions, which accounts for the differences in size measurements. Additionally, particle stability was not assessed immediately after formulation. At the moment of testing, the particle sizes had increased to 180 nm for LNP 1 and 160 nm for LNP 2, suggesting that some aggregation or swelling of the LNPs occurred. Nevertheless, since the size and potency of the sprayed LNPs were comparable to the non-sprayed controls, this swelling or aggregation does not seem to affect the overall comparison.

Additionally, we found that the particles are stable in pig cervicovaginal mucus but unstable in an in-house synthesized human cervicovaginal mucus simulant. The pH of cervicovaginal mucus of pigs (pH 6.5) and most mammals (pH 6.8) is more neutral compared to humans (pH 4.5) [39]. The cationic ionizable lipid of the LNP becomes positively charged at an acidic pH. Due to this change in charge, electrostatic repulsion between the charged lipids occurs, resulting in a decreased membrane packing and thus reduced stability [40]. This characteristic is also used to release the mRNA during acidification of the endosome. Therefore, for human intravaginal applications of sa-mRNA in LNPs, additional research is needed to overcome the acidic pH of the vaginal mucosa without influencing its protective characteristics or influencing endosomal escape.

## Conclusions

This study is the first to demonstrate mucosal uptake and expression of sa-mRNA encapsulated in lipid nanoparticles in pigs. Only local injection and not spraying of the particles in the nasal mucosa resulted in expression of the sa-mRNA, since mucociliary clearance likely removed the particles. Delivery optimization of the lipid nanoparticle can resolve this problem. In contrast, upon spraying, local expression was observed in the vaginal mucosa, which is a more relevant administration method compared to mucosal injection. The sa-mRNA could not only be expressed locally at the nasal and vaginal mucosa of the pig but also at the local draining lymph nodes, indicating their potential for immunization purposes.

## Supplementary Information

Below is the link to the electronic supplementary material.

Supplementary file1 (DOCX 25328 KB)

## Data Availability

The datasets described in this paper can be requested from the corresponding author on reasonable request.
