# Supplementary material for: Mucosal administration of lipid nanoparticles containing self-amplifying mRNA induces local uptake and expression in a pig model as a potential vaccination platform against STIs
Source: Drug Deliv Transl Res. 2025 Jun 11;16(1):330–46. doi: 10.1007/s13346-025-01877-x (PMC12682725; doi:10.1007/s13346-025-01877-x)
Supplement: Supplementary file 1 — Supplementary file1 (DOCX 25328 KB) [file 13346_2025_1877_MOESM1_ESM.docx]

Supplementary information

Mucosal administration of lipid nanoparticles containing self-amplifying mRNA induces local uptake and expression in a pig model as a potential vaccination platform against STIs

Ibe Van de Casteele^1,2^, Magalie Plovyt^1^, Magdalena Stuchlíková ^1^, Michiel Lanssens^1^, Ben Verschueren^1^, Quenten Denon^3^, Paul Van der Meeren^3^, Sean McCafferty^1,4^, Arlieke Gitsels ^2^, Pieter Cornillie^5^, Niek N. Sanders^4^, Aster Vandierendonck^1^, Katrien C. K. Poelaert^1*^, Daisy Vanrompay^2*^

**^1^** Ziphius NV, **B-9052 Zwijnaarde, Belgium**

^2^ Laboratory for Immunology and Animal Biotechnology, Department of Animal Sciences and Aquatic Ecology, Faculty of Bioscience Engineering, Ghent University, **B-9000 Ghent, Belgium**

^3^ Particle and Interfacial Technology group (PainT), Department of Green Chemistry and Technology, Faculty of Bioscience Engineering, Ghent University, **B-9000 Ghent, Belgium**

^4^ Laboratory of Gene Therapy, Department of Veterinary and Biosciences, Faculty of Veterinary Medicine, Ghent University, **B-9820 Merelbeke, Belgium**

^5^ Department of Morphology, Imaging, Orthopedics, Rehabilitation and Nutrition, Faculty of Veterinary Medicine, Ghent University, **B-9820 Merelbeke, Belgium**

^*^ Shared senior authors and corresponding authors: katrien.poelaert@ziphus.org & [daisy.vanrompay@ugent.be](mailto:daisy.vanrompay@ugent.be)


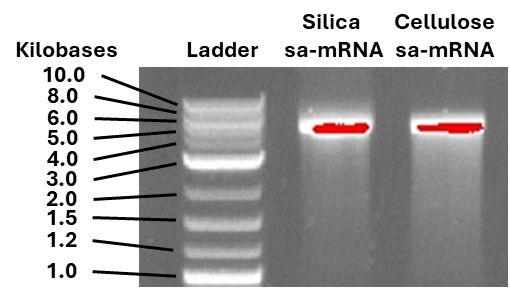


**Fig. S1 Bleach gel of the sa-mRNA used during the *in vitro* trials.** The quality of both silica purified and cellulose purified sa-mRNA was analyzed using the bleach gel. The 1kB plus DNA ladder was used to check the size of the sa-mRNA

**Fig. S2 Physicochemical characteristics of LNP 1 and LNP 2 used during the *in vitro* trials.** The average of three repeated measurements is given together with the standard deviation. The left y-axis presents the size of the particles, depicted by the blue columns and corresponding blue error bars. On the right y-axis, the zetapotential of the particles can be found, depicted by the black dots and black error bars


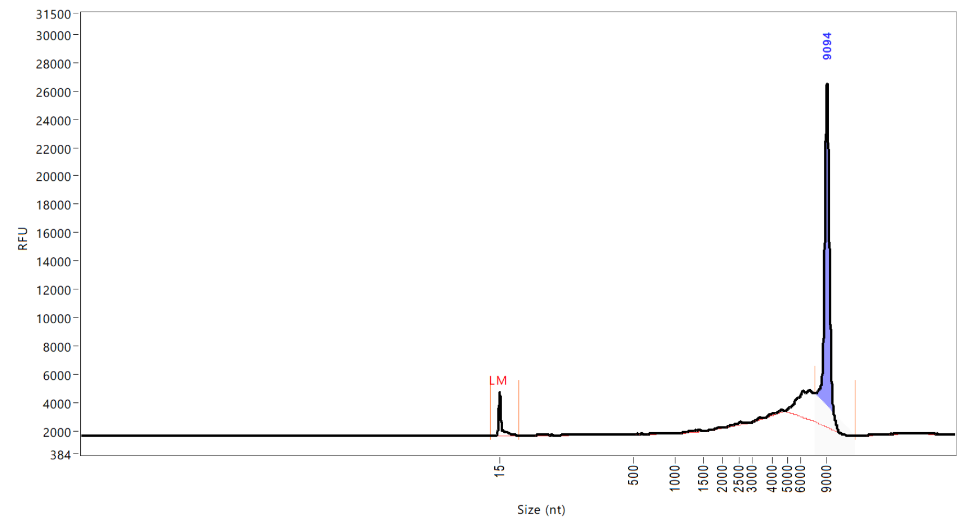


**Fig. S3 Fragment analyzer of the sa-mRNA used during the *in vivo* trials.** A fragment analyzer was used to assess the quality of the cellulose purified sa-mRNA used during the in vivo trial. On the X-axis, the size of the sa-mRNA is indicated based on a Lonza RNA ladder. The exact size of the sa-mRNA is given on top of the peak. The y-axis gives the signal intensity in relative fluorescent units (RFU). Smear analysis revealed a purity of 74.8% when comparing the signal in a 15% range from the peak to the total signal

**Fig. S4 Physicochemical characteristics of the LNPs used during the *in vivo* trials.** For each LNP, three repeated measurements were included. The average of these three measurements is given together with the standard deviation. On the left y-axis, the size of the particles can be found, depicted by the blue columns and the blue error bars. On the right y-axis, the zeta potential of the particles can be found, depicted by the black dots and the black error bars

**Fig. S5 *In vitro* transfection performed with the LNPs of the *in vitro* and two *in vivo* trials.** HeLa cells were transfected with sa-mRNA encapsulated in LNP 1 or LNP 2. After 24 hours, bioluminescent (a) and fluorescent signals (b) were measured. For the *in vitro* trial, four replicates were included in comparison to five replicates for the *in vivo* trial. These replicates are visualized together with the mean signal and the standard deviation. The average signal of the negative control is depicted with a dotted line. A cut-off for positive signals was established by adding two times the standard deviation and is depicted by a second dashed line. Significance is indicated by P<0.01 ** and P<0.001***

**Fig. S6 Plate layout and IVIS lumina III images from pig samples.** Two DiD fluorescently labelled LNP formulations were administered through mucosal injection or spraying at a dose of 15 µg sa-mRNA. a) The order of the tissues on the image can be found in the set-up depicted on top of the figure. b) Representative fluorescent images for each LNP and administration method are given. c) Representative bioluminescent images for each LNP and administration method are given
